# Genome-Wide Analysis and Exploration of WRKY Transcription Factor Family Involved in the Regulation of Shoot Branching in *Petunia*

**DOI:** 10.3390/genes13050855

**Published:** 2022-05-11

**Authors:** Huanyu Yao, Tianyin Yang, Jie Qian, Xinyi Deng, Lili Dong

**Affiliations:** College of Horticulture, Anhui Agricultural University, Hefei 230036, China; yaohuanyu@stu.ahau.edu.cn (H.Y.); yangtianyin@stu.ahau.edu.cn (T.Y.); qj97970809@126.com (J.Q.); xydeng2001@ahau.edu.cn (X.D.)

**Keywords:** *Petunia hybrida*, shoot branching, WRKY, expression analysis, PhWRKY71, functional analysis

## Abstract

The WRKY transcription factors (TFs) participate in various physiological, growth and developmental processes of plants. In our study, a total of 79 WRKY family members were identified and classified into three groups (Group I, Group IIa–e, and Group III) based on phylogenetic and conservative domain analyses. Conserved motif analysis showed that seven WRKYGQK domains changed. The promoter sequence analysis suggested that there were multiple stress- and hormone-related cis-regulatory elements in the promoter regions of *PhWRKY* genes. Expression patterns of *PhWRKYs* based on RNA-seq data revealed their diverse expression profiles in five tissues and under different treatments. Subcellular localization analysis showed that PhWRKY71 was located in the nucleus. In addition, overexpression of *PhWRKY71* caused a significant increase in branch number. This indicated that *PhWRKY71* played a critical role in regulating the shoot branching of *Petunia* *hybrida*. The above results lay the foundation for further revealing the functions of *PhWRKY* genes.

## 1. Introduction

WRKY, one of the ten largest transcription factor (TF) families in higher plants [1], is involved in regulating plant growth and development [2], senescence and disease resistance [3,4], hormone pathways [5], stress [6], and biosynthesis of secondary metabolites [7]. WRKYs possess conserved domains at the N-terminal and C-terminal. N-terminal has a highly conserved WRKYGQK sequence [8], and the C-terminal is composed of a zinc finger-like structure, CX_4-5_CX_22-23_HXH (C_2_H_2_) or CX_7_CX_23_HXC (C_2_HC) [9]. According to the number and the features of WRKY domains (WDs), WRKY TFs can be divided into three main groups (I, II, and III) [10]. Group I usually has two WDs, while the other two groups contain only one WRKY domain. Group I and II include the C_2_H_2_-type zinc finger, whereas Group III has the zinc finger structure of C_2_HC-type. Group II is further divided into five subgroups (IIa–e) according to different protein sequences [11].

Plant architecture is considered a particularly important trait in horticultural plants, whereas the branching pattern is a key component of plant architecture [12]. Generally, axillary shoot development consists of two stages: the initiation of axillary meristems (AM) in the axil and subsequent outgrowth of the axillary buds [13]. Whether these buds continue to grow to be lateral branches or become dormant mainly depends on endogenous and developmental signals and environmental factors [14]. Those factors may influence shoot branching via targeting different branching-related genes.

Studies in *Arabidopsis* *thaliana* showed that *WRKY71* increased excessive AM initiation and bud activity by positively regulating the transcription of *REGULATOR OF AXILLARY MERISTEMS 1* (*RAX1*), *RAX2, RAX3,* and negatively regulating auxin signaling [15]. In addition, overexpression of *WRKY8*, *WRKY28*, *WRKY48* and *WRKY57* driven by the *WRKY71* promoter or four copies of cauliflower mosaic virus (CaMV) 35S enhancers caused excessive lateral branches, resulting from much more AM initiation and elevated bud activities [15], which suggested that *WRKY* genes were important for plant morphological adaptation. With the completion of the whole genome mapping in various plants, WRKY genes have been identified and characterized, such as grape [16], watermelon [17], cucumber [18], and cabbage [19], etc.

*Petunia hybrida* Vilm is one of the most important ornamental crops. However, in its cultivation, it is necessary to manually remove the shoot apex to promote shoot branching so as to achieve the ornamental effect of flourishing flowers. This greatly increases the production expense. Therefore, the cultivation of new varieties with different branch types is one of the most important breeding directions in *P. hybrida*. In this study, the members of the WRKY family of *P. hybrida* were identified, and their phylogenetic relationship, exon–intron structure, conserved domains, cis-regulatory elements, gene expression pattern, subcellular localization and function of *PhWRKY71* were analyzed. This study provides a theoretical foundation for future research on the functions of *PhWRKY* genes in shoot branching.

## 2. Materials and Methods

### 2.1. Plant Materials

Petunia × hybrida cv ‘Mitchell Diploid’ seedlings were planted in pots (5 × 5 cm) and placed in a tissue culture incubator for cultivation at 24 ± 2 °C, a photoperiod of 16 h/8 h (light/dark), and a light intensity of 3500 LX.

### 2.2. Identification of PhWRKY Genes

The known *Petunia* WRKY proteins were used as the query sequences. The BLAST search was carried out on the *Petunia* genome website (https://solgenomics.net/tools/blast/, accessed on 2 October 2020), and the candidate sequences of WRKY proteins were obtained. These candidate sequences were further filtered by Pfam (http://pfam.janelia.org/, accessed on 2 October 2020) and SMART tools (http://smart.embl-heidelberg.de/, accessed on 2 October 2020). The repetitive sequences and sequences not containing WRKY domains were removed, and finally, the *P. axillaris WRKY* gene sequences were obtained. The prediction of the physical and chemical features of PhWRKY proteins, including molecular weight (MW), protein sequence length and isoelectric point (IP), were assessed with ExPASy (https://web.expasy.org/compute_pi/, accessed on 2 October 2020). To predict the subcellular localization of the identified PhWRKY proteins, WoLF PSORT (https://wolfpsort.hgc.jp/, accessed on 2 October 2020) was used to analyze the protein sequences.

### 2.3. Phylogenetic Tree Analysis of PhWRKYs

The WRKY protein sequences of *Petunia* and *Arabidopsis* were compared using MEGA X software, and a phylogenetic tree was constructed by the neighbor-joining method with a boot-strap value based on 1000 replicates. The *Arabidopsis* WRKY sequences were obtained from the UniProt protein database (https://www.uniprot.org/, accessed on 2 October 2020).

### 2.4. Analysis of Motifs and Gene Structure

The conserved motifs for each PhWRKY member were analyzed using MEME Suite version 5.0.5 (http://meme.nbcr.net/meme/, accessed on 4 October 2020). The gene structures of the *PhWRKYs* were determined by the online Gene Structure Display Server (GSDS) 2.0 software (http://gsds.cbi.pku.edu.cn/, accessed on 4 October 2020).

### 2.5. Analysis of the Cis-Elements in PhWRKY Promoters

The cis-elements in the promoter regions of *PhWRKY* genes were investigated by downloading the 1700 bp upstream sequences from the transcription start site of each putative *PhWRKY* gene. The promoter sequence analysis was then performed using PlantCARE databases (http://bioinformatics.psb.ugent.be/webtools/plantcare/html/, accessed on 10 October 2020).

### 2.6. Expression Analysis of PhWRKY Genes

Seventy-day-old seedlings of Petunia × hybrida cv ‘Mitchell Diploid’ were used as the experimental materials. For the tissue-specific expression experiment, roots, stems, leaves, axillary buds and flowers were harvested for RNA extraction.

For the treatment experiment, forty-day-old seedlings of Petunia × hybrida cv ‘Mitchell Diploid’ were divided into three groups. One group was used as control, the second group was decapitated and the third group was treated with 6-BA (50 μM) to the axillary buds at the top 4th nodes. After 6 h, the buds at the 1st nodes (decapitation) or 4th nodes (6-BA treatment) were harvested for RNA extraction. The RNA was placed in foam boxes containing dry ice and sent to Beijing Novogene Technology Co. Ltd. (Beijing, China) for transcriptome sequencing. Subsequently, Log2 based on the Fragments Per kb per Million reads (FPKM) value was used to create the heat map with the ClustVis tool (https://biit.cs.ut.ee/clustvis/, accessed on 15 October 2020).

### 2.7. Subcellular Localization

The coding regions of *PhWRKY71* were amplified by a polymerase chain reaction (PCR) with primers F (5′-3′): CCAAATCGACTCTAGAATGGCTGATGAACTAAGAA ATTT and R (5′-3′): CCACTAGTATTTAAATGTTCTTTTCCATCGAGACATT and then fused to the pSuper1300-eGFP plant expression vector by the homogenous recombination method. The fused plasmid pSuper1300-PhWRKY71-eGFP was then transformed into *Agrobacterium tumefaciens* GV3101, and the positive clones were selected for transient infiltration into leaves of *Nicotiana*. *benthamiana* for the subcellular localization assay. After at least 48 h of culture, the GFP fluorescence was observed under a confocal microscope (Olympus, Tokyo, Japan).

### 2.8. Overexpression of PhWRKY71 and Phenotype Analysis in Petunia

The 35S::PhWRKY71 was transformed to Petunia × hybrida cv ‘Mitchell Diploid’ according to the method reported by Guo et al. [20]. The expression level of *PhWRKY71* was detected by qRT-PCR. The number of branches with a bud length ≥10 mm was scored, and the height of the main stems was measured for phenotype analysis.

## 3. Results

### 3.1. Identification of the WRKY TFs in Petunia

In total, we identified 79 WRKY proteins through the *Petunia* genomic database in this study (Additional file: Table S1). The characteristics were analyzed, including protein length, MW and isoelectric point IP. As shown in Table S1, the length of PhWRKY proteins varied from 81 amino acids (aa) (PhWRKY54) to 741 aa (PhWRKY1), and the average length of protein sequence was 382 aa. The IP ranged from 4.8 (PhWRKY78) to 9.9 (PhWRKY54), and the MW ranged from 9577.91 Da (PhWRKY54) to 80,217.87 Da (PhWRKY1).

### 3.2. Phylogenetic Analysis and Classification of PhWRKY Proteins

**Figure 1:**
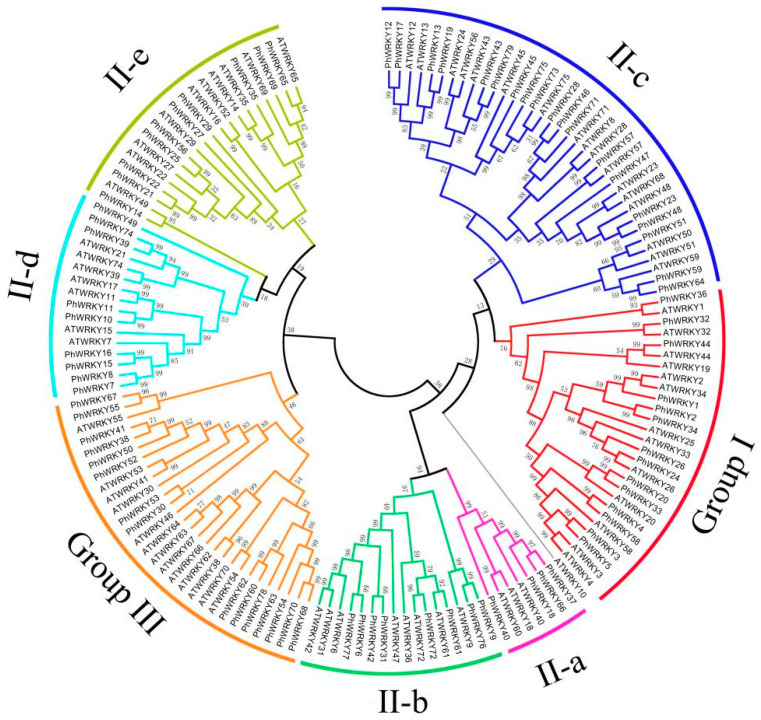
Phylogenetic tree of WRKYs from *Petunia* *hybrida* and *Arabidopsis* *thaliana*. The unrooted phylogenetic tree was constructed based on the WDs from *Petunia* and *Arabidopsis*. The names of groups (I, II a–e, and III) are shown outside of the circle. Branch lines of subtrees are colored for different WRKY subgroups.

To investigate the evolutionary relationships among the PhWRKY proteins, an unrooted phylogenetic tree was constructed with 71 AtWRKY and 79 PhWRKY proteins (Figure 1). Among the identified 79 PhWRKYs, there were 14 and 15 PhWRKYs belonging to Group I and III, respectively. Group II, as the largest group, contained 50 PhWRKYs, and was further divided into five subgroups as follows: Group IIa (4 PhWRKYs), Group IIb (8 PhWRKYs), Group IIc (19 PhWRKYs), Group IId (9 PhWRKYs), and Group IIe (10 PhWRKYs).

Multiple sequence alignment showed the existence of WRKYGQK and zinc-finger motifs in all identified PhWRKYs. The WRKY TFs have two conserved motifs, and the first one was WRKYGQK. In our study, three variations of the WRKYGQK sequence, WRKYGLK (PhWRKY50) in Group III, WRKYGMK (PhWRKY29) and WRKYGKK (PhWRKY51, PhWRKY59) in Group II, were found (Additional file: Figure S1). The other one was C2H2- or C2HC-type zinc finger motif. As shown in Figure S1, Group I was clustered by 14 PhWRKY proteins. Among them, PhWRKY34 lacked an intact WRKYGQK sequence in its N-terminal. The zinc-finger motif of the PhWRKYs in Group I belonged to the C_2_H_2_ type (C-X_4_-C-X_22_-H-X_1_-H). There were 50 members belonging to Group II, and 47 of them contained C-X_4-5_-C-X_23-27_-H-X_1_-H, while PhWRKY47, PhWRKY73 and PhWRKY76 all lacked a typical zinc-finger-like motif. The zinc-finger motif of Group III was C_2_HC type (C-X_5-7_-C-X_23-38_-H-X_1_-C), except PhWRKY41, which lacked an intact zinc-finger structure.

### 3.3. Gene Structure and Conserved Motif Analysis of PhWRKY Genes

**Figure 2:**
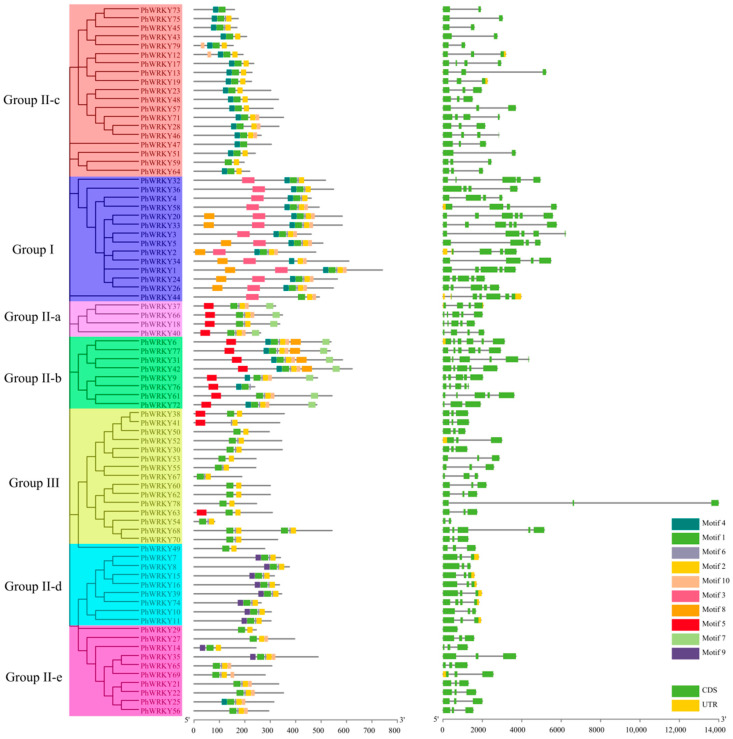
Sequence analysis and gene structure of PhWRKYs.

The gene structure of each *PhWRKY* gene was mapped here. As shown in Figure 2, the number of introns in *PhWRKY* genes ranged from zero to five. The majority of *PhWRKY* genes (41) had two introns; followed by 12 *PhWRKYs* with three introns; 12 *PhWRKYs* with four introns; seven *PhWRKYs* with one intron; six *PhWRKYs* with five introns; and *PhWRKY29* had no introns. As we can see from Figure 2, the most closely related members in the same group or subgroup presented a similar exon–intron structure. For instance, most of the *PhWRKY* genes in Group III contained two introns.

To obtain a better understanding of structural similarity and dissimilarity among different PhWRKY proteins, ten conserved motifs were identified. All PhWRKYs had the conserved heptapeptides WRKYGQK (Motif 1 or Motif 2), which is the basic feature of WRKYs. The conserved motifs were commonly found in specific groups. For example, the members of Group IIa included six conserved motifs (Motifs 1, 2, 5, 6, 7 and 10), and Group IIb and Group IId contained eight (Motifs 1, 2, 4, 5, 6, 7, 8 and 10) and four conserved motifs (Motifs 1, 2, 6 and 9), respectively. It was clear that some motifs specifically existed in one or more groups (subgroups). For instance, Motif 5 and Motif 7 existed in Group IIa and Group IIb members, and Motif 9 only existed in Group IId members, while Motif 8 was mainly present in Group I members.

### 3.4. Identification of Cis-Acting Elements in the Promoters of PhWRKY Genes

**Figure 3:**
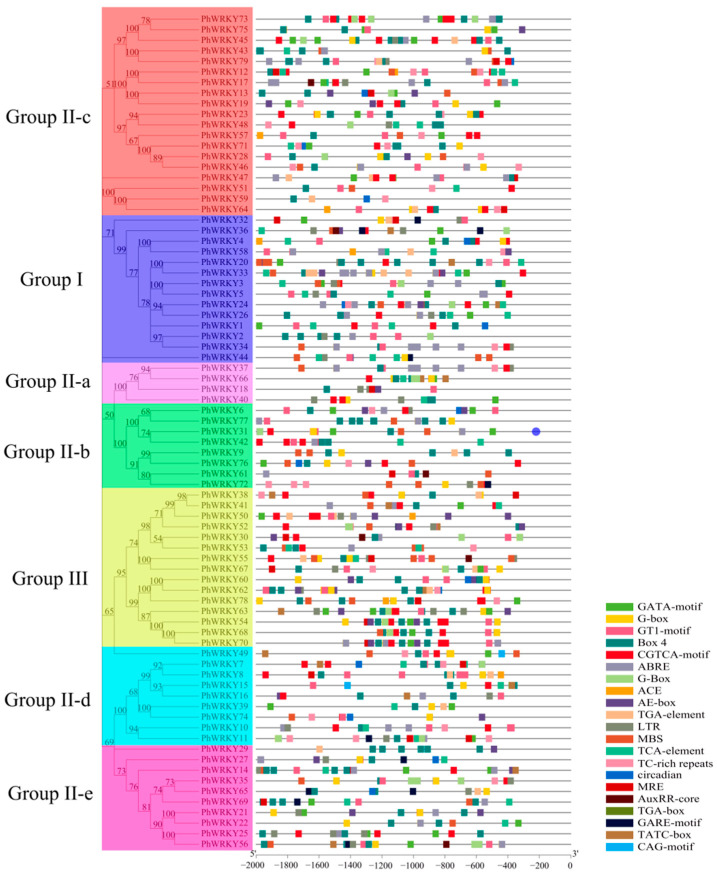
Types of cis-acting elements in promoter regions of *PhWRKY* genes.

The 1700 bp promoter sequences of the *PhWRKY* genes were extracted and submitted to the PlantCARE database to detect cis-acting elements. As depicted in Figure 3, in addition to a significant number of light response elements, there were a variety of hormone and stress response elements. The five hormone-related elements were MeJA-responsive element (CGTCA-motif), abscisic acid (ABA) response element (ABRE), auxin response elements (TGA-element, TGA-box, AuxRR-core), gibberellin (GA) response elements (GARE-motif, TATC-box) and salicylic acid (SA) reaction element (TCA-element). Four elements involved in stress were the defense and stress response element (TC-rich repeats), anaerobic induction (ARE), MYB binding sites (MBS) involved in drought induction, and cryogenic reaction element (LTR). The element associated with growth and development was the circadian rhythm regulatory element (circadian).

The light-responsive element (CATA-motif) and ABRE were the most abundant elements in the promoter regions of 79 *PhWRKYs*, with 67 promoters containing the two elements. CGTCA-motifs were found in 63 promoters. Forty-six gene promoters had the TCTA-box and GARE-motif. LTR, MBS, TCA-element and TGA-box were detected in 36, 39, 39, and 11 promoters of *PhWRKY* genes, respectively. The above promoter analysis revealed that the expression of *PhWRKY* genes might be associated with different environmental factors.

### 3.5. Analysis of Expression Patterns of PhWRKY Genes in Five Tissues

**Figure 4:**
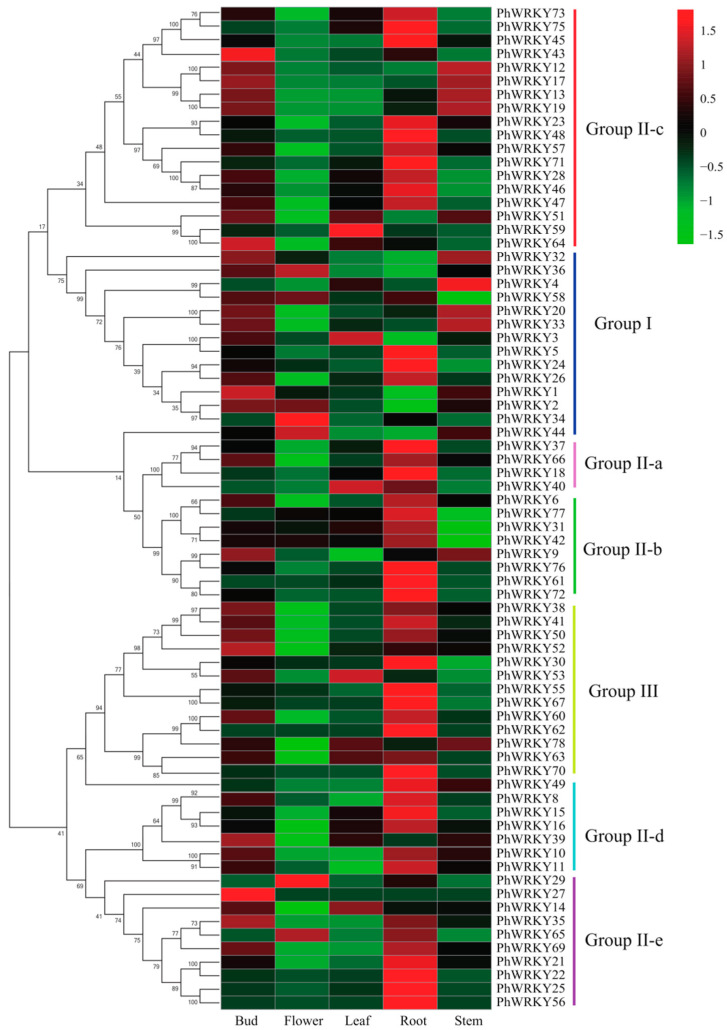
Expression analysis of *PhWRKY* genes in five tissues of *Petunia*.

To explore the potential functions of *PhWRKY* genes involved in plant growth and development, the expression profiles of *PhWRKYs* were analyzed based on transcriptome data from five tissues, including roots, stems, leaves, buds, and flowers (Figure 4). We detected the expression of 79 genes, while the expression of *PhWRKY7*, *PhWRKY54*, *PhWRKY68*, *PhWRKY74* and *PhWRKY79* were not detected in any of these five tissues, possibly due to the special expression patterns that cannot be examined in our libraries. Most of the *PhWRKY* genes (43) presented the highest expression level in the root, indicating that they might play an important role in *Petunia* root growth and development. The expression levels of *PhWRKY1*, *PhWRKY27*, *PhWRKY43*, *PhWRKY64* and *PhWRKY52* in buds were higher than that in any other tissue, revealing the potential functions of the five members in shoot branching.

In particular, we noticed that the expression patterns of *PhWRKY23*, *PhWRKY28*, *PhWRKY46*, *PhWRKY47*, *PhWRKY48*, *PhWRKY57* and *PhWRKY71* were similar to that of *WRKY71* [15]. That is, the expression level was highest in roots, followed by higher expression level in buds. The result suggested that these genes may have similar functions.

### 3.6. Response Analysis of PhWRKY Genes to Decapitation and 6-BA

**Figure 5:**
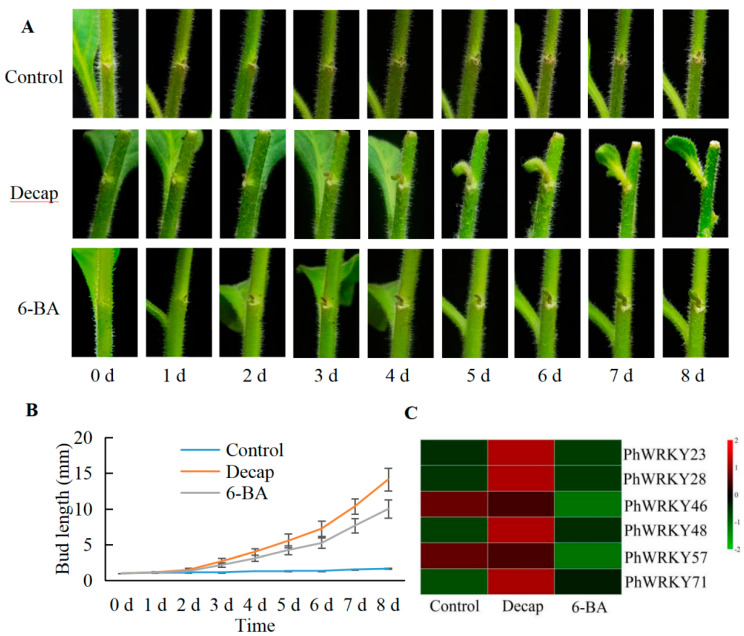
Response of Petunia × hybrida cv ‘Mitchell Diploid’ seedlings and *PhWRKY* genes to decapitation (decap) or 6-BA treatment. (**A**,**B**) Decapitation and application of 6-BA promoted the bud outgrowth of axillary buds. (**C**) Response of six *PhWRKY* genes to decapitation and 6-BA treatment.

In order to study the effects of decapitation and 6-BA on the growth of axillary buds of *Petunia*, the bud length at the first node or the fourth node was measured on different days after treatment. As can be seen from Figure 5, the average length of the buds increased from the second day and increased to 14.2 (decapitation) or 10.0 mm (6-BA) on the eighth day. However, no obvious bud growth of control was observed.

We further analyzed the expression characteristics of six *PhWRKY* genes. The expression levels of *PhWRKY23*, *PhWRKY28*, *PhWRKY48* and *PhWRKY71* increased dramatically after decapitation. However, 6-BA treatment did not significantly affect the expression levels of these four genes. The expression levels of *PhWRKY46* and *PhWRKY57* did not change significantly after decapitation, and the expression of *PhWRKY57* was not detected.

### 3.7. Subcellular Localization of PhWRKY71

**Figure 6:**
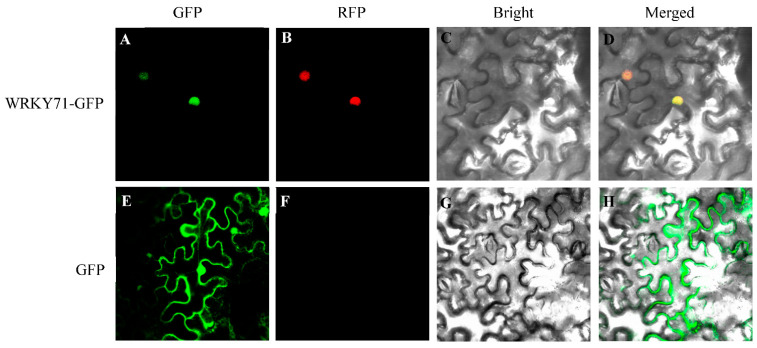
Subcellular localization of PhWRKY71 in *Nicotiana benthamiana* leaf epidermal cells. (**A**,**E**) Green fluorescence images of PhWRKY71-GFP protein and GFP (control). (**B**,**F**) Red fluorescence image of marker for nucleus localization. (**C**,**G**) Bright field images of PhWRKY71-GFP protein and control. (**D**,**H**) The merged images of PhWRKY71-GFP protein and control.

Since *PhWRKY71* can respond to decapitation and 6-BA treatment, and studies in *Arabidopsis* showed that *WRKY71* was involved in regulating shoot branching, we intend to study the function of the homologous gene of *WRKY71* in *Petunia*. To determine the subcellular localization of PhWRKY71, the coding sequence of *PhWRKY71* was fused to the *Green fluorescent protein* (*GFP*) gene driven by the CaMV 35S promoter. The constructed expression vector was used to perform a transient expression assay in *N. benthamiana* leaves. The GFP fluorescence of the control was distributed throughout the cell, while the green fluorescence of PhWRKY71-GFP and the red fluorescence of the nucleus-localization marker were both observed only in the nucleus (Figure 6). The subcellular localization pattern of PhWRKY71 was consistent with the characteristics of TFs.

### 3.8. Phenotypes of Transgenic Petunia Plants Overexpressing PhWRKY71

**Figure 7:**
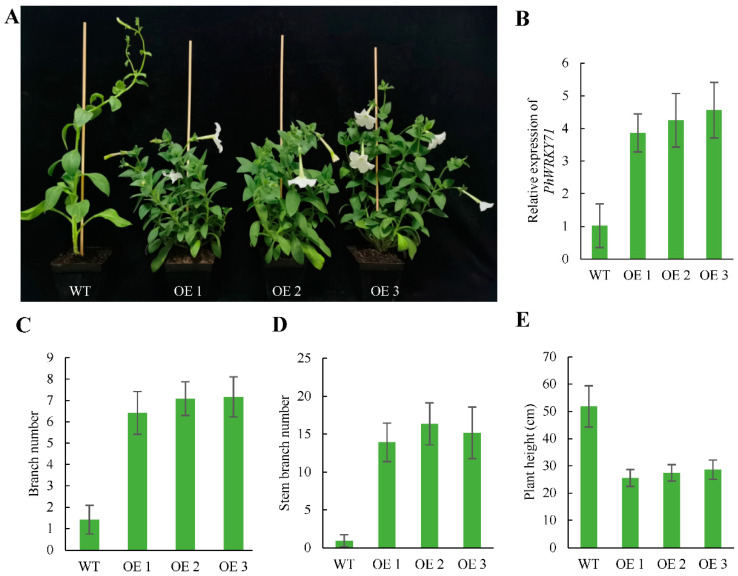
Phenotype and gene expression analysis of transgenic Petunia × hybrida cv ‘Mitchell Diploid’ plants overexpressing *PhWRKY71*. (**A**) Phenotypic comparisons of WT and transgenic plants overexpressing *PhWRKY71*. OE 1, OE 2 and OE 3 represent different lines overexpressing *PhWRKY71*. (**B**) The expression level of *PhWRKY71* was assayed by quantitative real-time–PCR (qRT-PCR). Detection of *PhACTIN* was used as the control. Three samples with 10 plantlets for each were averaged (±SE). (**C**) The basal branch number for each is shown (*n* = 12). (**D**) The stem branch number for each is shown (*n* = 12). (**E**) The height of the main stem for each is shown (*n* = 12).

The 35S::PhWRKY71 was transformed into *Petunia*, and a total of 14 independent transgenic T0 lines were obtained. In the T2 generation, three independent transgenic lines (OE 1, OE 2 and OE 3), with higher expression levels and obvious phenotypic differences, were selected for further phenotype analysis. We observed that overexpression of *PhWRKY71* induced more branches accompanied by a semi-dwarf phenotype (Figure 7A). The *PhWRKY71*-overexpression (PhWRKY71-OE) lines all displayed a marked increase in branching (Figure 7C), including basal branches and stem branches. On average, the number of basal branches from lines OE 1, OE 2 and OE 3 was 6.4, 7.1 and 7.2, respectively, while the control had 1.4 branches. The number of stem branches from lines OE 1, OE 2 and OE 3 was 13.9, 16.3 and 15.2, respectively, compared to the control with 0.9 branches. Furthermore, the average plant height of the three PhWRKY71-OE lines was 25.5, 27.4 and 28.6 cm, respectively, whereas that of control plants reached 51.8 cm on average (Figure 7D). Thus, the average plant height in the three PhWRKY71-OE lines was decreased by 47%. Taken together, our results indicated that *PhWRKY71* was a crucial regulator for shoot branching and plant height.

## 4. Discussion

The WRKY TF family, as one of the largest families of plant transcriptional regulators, has been identified in various species. Although some research on *Petunia* WRKYs has been reported [21], characterization and function analysis of PhWRKYs were still insufficient. In the present study, 79 *PhWRKY* genes were systematically characterized in *Petunia* for the first time. The number was higher than that in *Dianthus caryophyllus* (53) [22], and *Cucumis Melo* L. (57) [23] but lower than that in *Triticum aestivum* (177) [24] and *Zea mays* (119) [11], suggesting a distinct evolutionary expansion of WRKY genes among different plant species. PhWRKYs were classified into three major groups (I, II, III) based on the conserved domains, and Group I, II and III contained 14, 50 and 15 members, respectively. The composition of the WRKY gene family drastically varies among different plant species, for example, previous research has demonstrated that Group I houses the largest number of WRKYs in poplar [25], while Group III is largest in rice [26]. Therefore, we speculate that numerous Group II members in *Petunia* implies more gene duplications during the course of evolution.

In our study, almost all of the PhWRKY proteins contained the WRKYGQK domain, whereas some variations, such as WRKYGLK, WRKYGMK and WRKYGKK, were also found. Slight variations of the WRKYGQK domain have also been reported in other plants. For instance, there were nine changed SlWRKY domains in tomato [27], and six changed VvWRKY domains in *Vitis vinifera* [28]. Previous studies have shown that the WRKYGQK domain can bind to W-box cis-elements in the promoter region to activate the transcription of downstream target genes [1], while the variation of the WRKYGQK domain may affect the DNA-binding specificity. Therefore, further work is required to investigate the molecular function and binding specificity of each protein with motif variations. 

The MWs and IPs of the PhWRKY proteins showed large variations, likely due to their different functions in plant growth and development. The motif analysis of PhWRKY proteins demonstrated that PhWRKY proteins that clustered into the same phylogenetic clade showed highly similar motif distributions and exon–intron structures, implying the functional similarity. Most of the *PhWRKY* genes had two introns, as commonly found in other plants, such as sesame [29], chickpea [30], pear [31] and cassava [32]. Overall, the position and number of introns in the same group showed a good similarity. For example, the Group I members contained four to six introns, whereas most of the Group III members consisted of two introns. In addition, the distribution proportion of introns in different species of the same family was also similar. For example, there were two introns in tomato and *Petunia*, accounting for 60% and 52% of the WRKY family, respectively, followed by three introns (13.5% in tomato and 9% in *Petunia*). About 2.5% of tomato WRKYs and 1.2% of *Petunia* WRKYs were intron-free. These results indicated that the distribution of introns among different species was conserved to some extent.

In our study, the expression profile for each *PhWRKY* gene in five different tissues was revealed, providing valuable data for further research. As can be seen from the evolutionary tree, PhWRKY23, PhWRKY28, PhWRKY46, PhWRKY48, PhWRKY57, PhWRKY71 were closely related to WRKY8, WRKY28, WRKY48 and WRKY57. Studies showed that *WRKY8*, *WRKY28*, *WRKY48* and *WRKY57* had similar expression patterns to *WRKY71*/*EXCESSIVE BRANCHES1* (*EXB1*). The ectopic expression of *WRKY8*, *WRKY28*, *WRKY23*, *WRKY48* and *WRKY57* all caused excessive branching [15,33]. Therefore, we speculate that *PhWRKY23*, *PhWRKY28*, *PhWRKY46*, *PhWRKY48*, *PhWRKY57* and *PhWRKY71* are involved in shoot branching in *Petunia*. Tissue expression analysis showed that the expression levels of these genes were highest in roots, implying that these genes not only participated in shoot branching but also played an important role in root growth and development. Moreover, we found that decapitation promoted the obvious up-regulation of *PhWRKY23*, *PhWRKY28*, *PhWRKY48* and *PhWRKY71*, indicating that decapitation may partially regulate the expression of these genes, resulting in bud outgrowth.

Overexpression of *PhWRKY71* caused a remarkable increase in the total number of lateral branches. Not only the number of branches at the base increased notably, but also the branches on the stem were significantly more than those in the control. This indicates that the overexpression of *PhWRKY71* greatly weakens the apical dominance of *Petunia*. At the same time, we noticed that the plant height of transgenic lines decreased, which was similar to the phenotype of plants overexpressing *WRKY71* in *Arabidopsis* [15], suggesting the functional conservation of the homologous gene. The function of *PhWRKY71* is also similar to that of most genes that regulate shoot branching, implying there are some relationships between plant height and shoot branching. In addition, how *PhWRKY71* regulates branch development of *Petunia* and what its direct downstream target genes are need to be further studied.

## 5. Conclusions

Genome-wide analysis of phylogenetic relationships, intron/exon structures, motif compositions, and expression characteristics of *PhWRKY* genes was carried out in this study. A total of 79 WRKY members from *Petunia* were identified. Gene expression analysis revealed that most of the *PhWRKY* genes had tissue-specific expression patterns, and some of them were speculated to be involved in bud development, consistent with the molecular function predictions based on phylogenetic analysis. Some *PhWRKY* genes could respond to decapitation, suggesting that decapitation may cause axillary bud growth partly by affecting the expression levels of these *PhWRKY* genes. PhWRKY71 was shown to be localized in the nucleus. Overexpression of *PhWRKY71* caused an excessive increase in branch number and a significant decrease in plant height, indicating an important role in regulating shoot branch development.

## Data Availability

The RNA-seq data generated during the current study were obtained from the NCBI (ID: PRJNA836568).

## Supplementary Materials

The following supporting information can be downloaded at: https://www.mdpi.com/article/10.3390/genes13050855/s1, Figure S1: Comparison of WRKY domain sequences from PhWRKY proteins, Table S1: Protein information of WRKY TF in *Petunia*.
