# Supplementary material for: Genome-Wide Analysis and Exploration of WRKY Transcription Factor Family Involved in the Regulation of Shoot Branching in Petunia
Source: Genes (Basel). 2022 May 11;13(5):855. doi: 10.3390/genes13050855 (PMC9141166; doi:10.3390/genes13050855)
Supplement: Supplementary file 1 [file genes-13-00855-s001.zip › Table S1.pdf]

Accession number	Gene name	Subcellular localization	Number of amino acids	Isoelectric point	Molecular weight	Molecular formula
Peinf101Scf00055g17013.1	PhWRKY1	Nucleus	741	5.96	80217.87	$C_{3444}H_{5334}N_{1026}O_{1146}S_{24}$
Peinf101Scf01201g03016.1	PhWRKY2	Nucleus	479	7.62	53467.68	$C_{2351}H_{3652}N_{660}O_{741}S_{14}$
Peinf101Scf00231g01029.1	PhWRKY3	Nucleus	461	6.41	50554.87	$C_{2182}H_{3421}N_{641}O_{717}S_{14}$
Peinf101Scf01969g05034.1	PhWRKY4	Nucleus	461	6.84	51786.12	$C_{2254}H_{3544}N_{632}O_{722}S_{23}$
Peinf101Scf00898g01016.1	PhWRKY5	Nucleus	507	6.84	55393.85	$C_{2375}H_{3727}N_{703}O_{799}S_{15}$
Peinf101Scf00923g03009.1	PhWRKY6	Nucleus	539	6.56	60169.56	$C_{2598}H_{4122}N_{766}O_{826}S_{27}$
Peinf101Scf01179g02021.1	PhWRKY7	Nucleus	340	9.64	37933.63	$C_{1638}H_{2621}N_{497}O_{515}S_{13}$
Peinf101Scf00949g01005.1	PhWRKY8	Nucleus	375	9.59	41547.69	$C_{1794}H_{2871}N_{537}O_{569}S_{15}$
Peinf101Scf00255g03015.1	PhWRKY9	Nucleus	486	6.29	53512.31	$C_{2282}H_{3635}N_{679}O_{766}S_{21}$
Peinf101Scf00276g07026.1	PhWRKY10	Nucleus	304	9.62	33135.67	$C_{1440}H_{2318}N_{426}O_{443}S_{14}$
Peinf101Scf00146g01031.1	PhWRKY11	Nucleus	303	9.86	32857.45	$C_{1424}H_{2312}N_{434}O_{433}S_{13}$
Peinf101Scf01786g02023.1	PhWRKY12	Nucleus	193	9.26	21957.43	$C_{946}H_{1483}N_{291}O_{298}S_{8}$
Peinf101Scf00239g16004.1	PhWRKY13	Nucleus	228	9.14	26300.67	$C_{1156}H_{1809}N_{337}O_{349}S_{9}$
Peinf101Scf01724g02031.1	PhWRKY14	Nucleus	244	4.94	27492.43	$C_{1206}H_{1854}N_{320}O_{398}S_{9}$
Peinf101Scf00887g05031.1	PhWRKY15	Nucleus	316	9.59	35256.13	$C_{1525}H_{2480}N_{446}O_{480}S_{16}$
Peinf101Scf00748g00019.1	PhWRKY16	Nucleus	336	9.38	37424.77	$C_{1631}H_{2634}N_{468}O_{503}S_{18}$
Peinf101Scf00883g11015.1	PhWRKY17	Nucleus	235	6.89	26740.26	$C_{1137}H_{1757}N_{353}O_{376}S_{11}$
Peinf101Scf00256g12020.1	PhWRKY18	Nucleus	337	6.71	37664.86	$C_{1619}H_{2565}N_{481}O_{530}S_{13}$
Peinf101Scf01468g07025.1	PhWRKY19	Nucleus	226	8.97	25723.02	$C_{1125}H_{1746}N_{320}O_{345}S_{14}$
Peinf101Scf00022g02026.1	PhWRKY20	Nucleus	583	6.42	62857.70	$C_{2702}H_{4268}N_{792}O_{894}S_{22}$
Peinf101Scf01997g00005.1	PhWRKY21	Nucleus	333	6.17	36939.62	$C_{1587}H_{2445}N_{463}O_{526}S_{16}$
Peinf101Scf00355g03016.1	PhWRKY22	Nucleus	352	5.68	38910.97	$C_{1676}H_{2624}N_{486}O_{557}S_{13}$
Peinf101Scf00244g18025.1	PhWRKY23	Nucleus	302	6.39	33685.60	$C_{1469}H_{2288}N_{406}O_{475}S_{14}$
Peinf101Scf00450g00007.1	PhWRKY24	Nucleus	564	6.65	62355.24	$C_{2685}H_{4145}N_{775}O_{904}S_{19}$
Peinf101Scf00962g16030.1	PhWRKY25	Nucleus	314	5.11	35577.78	$C_{1561}H_{2406}N_{416}O_{502}S_{17}$
Peinf101Scf00782g10028.1	PhWRKY26	Nucleus	548	8.13	60836.47	$C_{2614}H_{4030}N_{762}O_{881}S_{19}$

Peinf101Scf01632g07037.1	PhWRKY27	Nucleus	396	5.28	44534.07	$C_{1972}H_{3056}N_{534}O_{613}S_{15}$
Peinf101Scf00665g17027.1	PhWRKY28	Nucleus	334	7.56	37815.72	$C_{1639}H_{2510}N_{466}O_{535}S_{16}$
Peinf101Scf03679g05006.1	PhWRKY29	Nucleus	245	8.14	28577.68	$C_{1237}H_{1922}N_{366}O_{396}S_{10}$
Peinf101Scf01632g03025.1	PhWRKY30	Nucleus	347	5.66	38612.28	$C_{1665}H_{2542}N_{480}O_{553}S_{15}$
Peinf101Scf00039g14008.1	PhWRKY31	Nucleus	584	6.02	63221.31	$C_{2707}H_{4285}N_{793}O_{897}S_{29}$
Peinf101Scf00614g00012.1	PhWRKY32	Nucleus	517	7.01	56800.34	$C_{2423}H_{3911}N_{725}O_{808}S_{21}$
Peinf101Scf00071g01019.1	PhWRKY33	Nucleus	583	6.24	63112.70	$C_{2699}H_{4257}N_{801}O_{903}S_{23}$
Peinf101Scf00519g04010.1	PhWRKY34	Nucleus	609	5.90	67324.53	$C_{2887}H_{4546}N_{850}O_{958}S_{26}$
Peinf101Scf00423g15008.1	PhWRKY35	Nucleus	488	5.80	52907.89	$C_{2257}H_{3526}N_{664}O_{773}S_{18}$
Peinf101Scf01775g01023.1	PhWRKY36	Nucleus	549	5.83	59467.43	$C_{2519}H_{4054}N_{752}O_{878}S_{17}$
Peinf101Scf01889g05028.1	PhWRKY37	Nucleus	321	8.35	35691.89	$C_{1534}H_{2460}N_{440}O_{511}S_{14}$
Peinf101Scf02382g03038.1	PhWRKY38	Nucleus	355	5.45	40136.18	$C_{1758}H_{2676}N_{480}O_{576}S_{12}$
Peinf101Scf00571g28006.1	PhWRKY39	Nucleus	345	9.63	38680.77	$C_{1660}H_{2679}N_{517}O_{514}S_{18}$
Peinf101Scf01580g05011.1	PhWRKY40	Nucleus	262	6.08	29667.10	$C_{1284}H_{2030}N_{366}O_{420}S_{11}$
Peinf101Scf00962g23035.1	PhWRKY41	Nucleus	337	5.14	38214.23	$C_{1665}H_{2536}N_{460}O_{542}S_{17}$
Peinf101Scf01579g02011.1	PhWRKY42	Nucleus	622	6.16	67710.50	$C_{2876}H_{4516}N_{872}O_{967}S_{29}$
Peinf101Scf00962g16031.1	PhWRKY43	Nucleus	207	9.04	23751.12	$C_{1041}H_{1628}N_{302}O_{306}S_{15}$
Peinf101Scf00457g28014.1	PhWRKY44	Nucleus	493	9.19	55044.51	$C_{2362}H_{3789}N_{711}O_{768}S_{19}$
Peinf101Scf01192g04049.1	PhWRKY45	Nucleus	169	9.40	19389.75	$C_{840}H_{1324}N_{250}O_{263}S_{8}$
Peinf101Scf01109g09006.1	PhWRKY46	Nucleus	264	6.66	29317.28	$C_{1253}H_{1939}N_{357}O_{425}S_{16}$
Peinf101Scf01169g01016.1	PhWRKY47	Nucleus	304	5.11	33910.75	$C_{1483}H_{2275}N_{403}O_{478}S_{16}$
Peinf101Scf00078g04017.1	PhWRKY48	Nucleus	332	6.55	36954.85	$C_{1603}H_{2490}N_{454}O_{528}S_{12}$
Peinf101Scf00985g06014.1	PhWRKY49	Nucleus	278	5.24	31222.74	$C_{1368}H_{2108}N_{364}O_{447}S_{13}$
Peinf101Scf02382g03051.1	PhWRKY50	Nucleus	296	7.06	32490.35	$C_{1409}H_{2222}N_{394}O_{460}S_{14}$
Peinf101Scf00381g17007.1	PhWRKY51	Nucleus	241	6.58	27812.13	$C_{1240}H_{1863}N_{329}O_{378}S_{12}$
Peinf101Scf01602g02010.1	PhWRKY52	Nucleus	345	6.11	38895.74	$C_{1713}H_{2700}N_{466}O_{545}S_{11}$
Peinf101Scf00122g02023.1	PhWRKY53	Nucleus	244	6.02	27394.87	$C_{1189}H_{1880}N_{328}O_{384}S_{15}$
Peinf101Scf13097g00003.1	PhWRKY54	Nucleus	81	9.90	9577.91	$C_{422}H_{661}N_{127}O_{121}S_{4}$
Peinf101Scf00339g01023.1	PhWRKY55	Nucleus	243	6.13	27816.09	$C_{1214}H_{1863}N_{347}O_{378}S_{14}$

Peinf101Scf02382g04035.1	PhWRKY56	Nucleus	294	6.41	33143.03	$C_{1460}H_{2266}N_{396}O_{466}S_{10}$
Peinf101Scf01631g01036.1	PhWRKY57	Nucleus	311	5.76	34040.32	$C_{1462}H_{2285}N_{425}O_{496}S_{9}$
Peinf101Scf01214g04036.1	PhWRKY58	Nucleus	492	6.60	54321.64	$C_{2362}H_{3695}N_{673}O_{758}S_{21}$
Peinf101Scf02876g00007.1	PhWRKY59	Nucleus	197	8.66	22945.50	$C_{1011}H_{1544}N_{274}O_{322}S_{8}$
Peinf101Scf00191g46015.1	PhWRKY60	Nucleus	300	6.12	34348.16	$C_{1494}H_{2322}N_{418}O_{487}S_{13}$
Peinf101Scf02160g04021.1	PhWRKY61	Nucleus	543	6.87	59889.60	$C_{2560}H_{4097}N_{745}O_{863}S_{24}$
Peinf101Scf02687g00042.1	PhWRKY62	Nucleus	300	6.42	34628.61	$C_{1516}H_{2350}N_{424}O_{483}S_{12}$
Peinf101Scf00782g02035.1	PhWRKY63	Nucleus	308	5.64	34744.77	$C_{1512}H_{2374}N_{418}O_{495}S_{13}$
Peinf101Scf01619g01001.1	PhWRKY64	Nucleus	219	6.82	24944.33	$C_{1082}H_{1635}N_{307}O_{355}S_{10}$
Peinf101Scf00102g02002.1	PhWRKY65	Nucleus	306	6.05	34171.81	$C_{1454}H_{2294}N_{420}O_{496}S_{18}$
Peinf101Scf01142g06005.1	PhWRKY66	Nucleus	348	8.59	38505.78	$C_{1633}H_{2653}N_{485}O_{561}S_{14}$
Peinf101Scf02687g00049.1	PhWRKY67	Nucleus	188	5.15	20896.28	$C_{914}H_{1395}N_{249}O_{292}S_{11}$
Peinf101Scf00192g00019.1	PhWRKY68	Nucleus	543	5.58	61394.29	$C_{2668}H_{4118}N_{724}O_{877}S_{32}$
Peinf101Scf00442g03028.1	PhWRKY69	Nucleus	280	5.63	30462.63	$C_{1307}H_{2031}N_{371}O_{440}S_{15}$
Peinf101Scf00192g00020.1	PhWRKY70	Nucleus	329	5.77	37138.58	$C_{1610}H_{2515}N_{441}O_{526}S_{21}$
Peinf101Scf00040g09006.1	PhWRKY71	Nucleus	352	6.78	39578.71	$C_{1722}H_{2629}N_{487}O_{557}S_{16}$
Peinf101Scf00974g18005.1	PhWRKY72	Nucleus	483	6.35	53519.64	$C_{2309}H_{3688}N_{656}O_{771}S_{17}$
Peinf101Scf00889g03041.1	PhWRKY73	Nucleus	159	9.08	18148.20	$C_{784}H_{1224}N_{228}O_{253}S_{8}$
Peinf101Scf01061g09001.1	PhWRKY74	Nucleus	264	9.48	29326.45	$C_{1255}H_{2043}N_{393}O_{384}S_{17}$
Peinf101Scf00052g06026.1	PhWRKY75	Nucleus	174	9.27	20317.78	$C_{876}H_{1369}N_{257}O_{277}S_{12}$
Peinf101Scf00049g07032.1	PhWRKY76	Nucleus	239	8.85	27065.47	$C_{1155}H_{1873}N_{341}O_{380}S_{14}$
Peinf101Scf01200g03007.1	PhWRKY77	Nucleus	536	6.36	58740.76	$C_{2518}H_{4009}N_{743}O_{820}S_{29}$
Peinf101Scf00339g02023.1	PhWRKY78	Nucleus	246	4.80	28302.63	$C_{1263}H_{1933}N_{321}O_{402}S_{8}$
Peinf101Scf02382g04034.1	PhWRKY79	Nucleus	154	9.41	17774.21	$C_{782}H_{1242}N_{228}O_{234}S_{6}$
