# Supplementary figures and images for: Genome-Wide Analysis and Exploration of WRKY Transcription Factor Family Involved in the Regulation of Shoot Branching in Petunia
Source: Genes (Basel). 2022 May 11;13(5):855. doi: 10.3390/genes13050855 (PMC9141166; doi:10.3390/genes13050855)

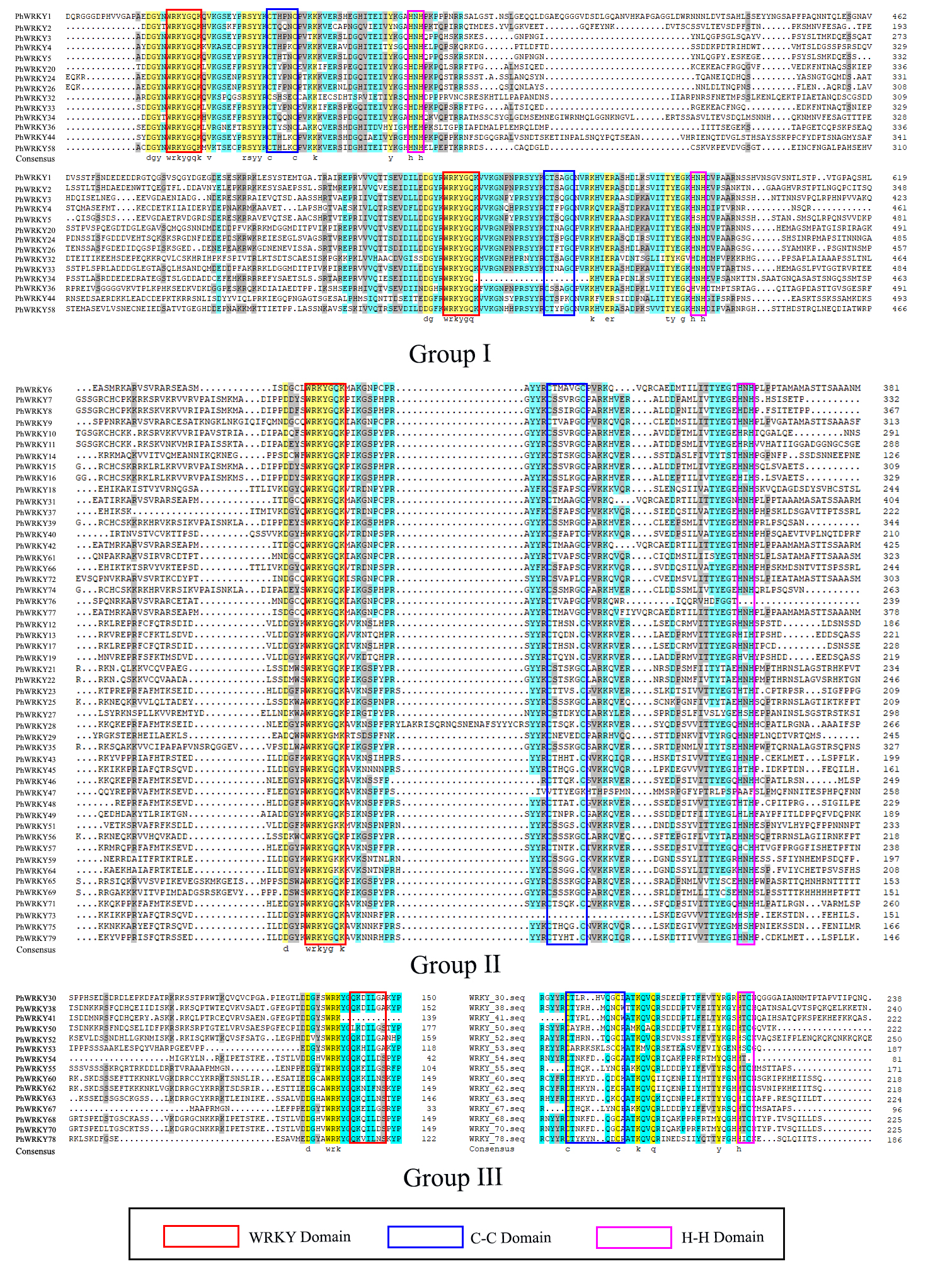

Supplement: Supplementary file 1 [file genes-13-00855-s001.zip › Figure S1.jpg]
